# Supplementary material for: Reconciling Mining with the Conservation of Cave Biodiversity: A Quantitative Baseline to Help Establish Conservation Priorities
Source: PLoS One. 2016 Dec 20;11(12):e0168348. doi: 10.1371/journal.pone.0168348 (PMC5173368; doi:10.1371/journal.pone.0168348)
Supplement: S1 Dataset — (ZIP) [file pone.0168348.s002.zip › Taxa/Serra Norte/SN_2013/N4E/Lista N4E-78.pdf]

## CAVIDADE N4E-0078

| Classe | Ordem | Fam/Outros | Gên/Outros | Espécie | S-U | U-S | Zon. |
|---|---|---|---|---|---|---|---|
| Annelida | Oligochaeta | | | jovem | | 4 | E |
| Arachnida | Acari | Anystidae | *Erythracarus* | sp.1 | | X | E |
| Arachnida | Acari | Neothyridae | *Diplothyrus* | sp.1 | | X | P |
| Arachnida | Acari | Veigaiidae | | sp.1 | X | | E |
| Arachnida | Amblypygi | Charinidae | *Charinus* | sp.1 | | 1 | P |
| Arachnida | Amblypygi | Phrynidae | *Heterophrynus* | *longicornis* | 1 | | P |
| Arachnida | Araneae | Barychelidae | | jovem | 1 | 1 | E |
| Arachnida | Araneae | Ctenidae | | jovem | 1 | | |
| Arachnida | Araneae | Drymusidae | *Drymusa* | *spelunca* | | X | P |
| Arachnida | Araneae | Oonopidae | | jovem | | X | E |
| Arachnida | Araneae | Pholcidae | *Mesabolivar* | *aurantiacus* | X | | |
| Arachnida | Araneae | Pholcidae | *Mesabolivar* | sp.1 | X | | E |
| Arachnida | Araneae | Pisauridae | | jovem | 1 | | E |
| Arachnida | Araneae | Prodidomidae | | jovem | | X | P |
| Arachnida | Araneae | Salticidae | | sp.4 | X | | E |
| Arachnida | Araneae | Theridiidae | *Theridion* | sp.4 | X | X | E/P |
| Arachnida | Araneae | Theridiosomatidae | *Plato* | sp.1 | X | X | E/P |
| Arachnida | Palpigradi | Eukoeneniidae | *Allokoenenia* | sp.1 | | X | P |
| Arachnida | Pseudoscorpiones | Olpiidae | | jovem | X | | E |
| Chilopoda | Geophilomorpha | Mecistocephalidae | *Mecistocephalus* | sp.1 | | 1 | E |
| Diplopoda | Polydesmida | Cyrtodesmidae | | sp.1 | X | | E |
| Diplopoda | Polydesmida | Fuhrmannodesmidae | | sp.1 | | X | P |
| Entognatha | Collembola | Paronellidae | | sp.1 | X | X | E/P |
| Entognatha | Collembola | Sminthuroidea | | sp.2 | | X | E |
| Insecta | Blattodea | Nocticolidae | | sp.1 | 2 | 1 | P |
| Insecta | Coleoptera | Chrysomelidae | | sp.17 | | X | E |
| Insecta | Coleoptera | Staphylinidae | Pselaphinae | sp.2 | X | X | E/P |
| Insecta | Diptera | Chironomidae | | sp. | X | | E/P |
| Insecta | Diptera | Phoridae | | sp. | | X | E |
| Insecta | Diptera | Tipulidae | Tipulinae | sp. | X | | E/P |
| Insecta | Homoptera | Cixiidae | | jovem | X | X | E/P |
| Insecta | Hymenoptera | Formicidae | *Solenopsis* | sp.1 | | X | P |
| Insecta | Hymenoptera | Formicidae | *Hypoponera* | sp.1 | | X | E |
| Insecta | Hymenoptera | Formicidae | *Odontomachus* | sp.1 | | X | E |
| Insecta | Hymenoptera | Formicidae | *Paratrechina* | sp.2 | X | X | E/P |
| Insecta | Hymenoptera | Formicidae | *Solenopsis* | sp.1 | X | X | E/P |
| Insecta | Lepidoptera | Noctuoidea | | sp.5 | 1 | | E |
| Insecta | Lepidoptera | Noctuoidea | | sp.6 | 1 | | E |
| Insecta | Orthoptera | Phalangopsidae | *Paraclodes* | jovem | 1 | | E |
| Insecta | Orthoptera | Phalangopsidae | *Phalangopsis* | sp. | 19 | 12 | P |
| Gastropoda | Pulmonata | Bulimulidae | *Naesiotus* | sp. | X | X | E/P |
| Gastropoda | Pulmonata | Systrophiidae | *Happia* | sp. | X | X | E/P |
| Malacostraca | Isopoda | Philosciidae | | sp.1 | | X | E |
| Malacostraca | Isopoda | Philosciidae | | sp.2 | X | | E |
| Malacostraca | Isopoda | Platyarthridae | | sp.1 | | X | E |
| Symphyla | | Scutigerellidae | | sp. | X | X | E/P |
| Amphibia | Anura | Strabomantidae | *Pristimantis* | cf. *fenestratus* | 2 | 1 | E/P |
| Mammalia | Chiroptera | Furipteridae | *Furipterus* | *horrens* | 1 | | P |
